# Criminal recidivism factors among offenders undergoing forensic psychiatric evaluation

**DOI:** 10.1192/j.eurpsy.2023.1872

**Published:** 2023-07-19

**Authors:** R. Felhi, G. Hamdi, S. Chebli, R. Ridha

**Affiliations:** Razi hospital, Manouba

## Abstract

**Introduction:**

Forensic studies often found high rates of major psychiatric and substance use disorders among inmates. These factors are linked to criminality in general, however, their involvement in recidivism among this population seems to be more important to determine.

**Objectives:**

The aim of this study is to determinesociodemographic, psychiatric and forensicfactors of criminal recidivism among prisoners

**Methods:**

We studied the medical files of all the offenders referred to the forensic psychiatry unit in Razi hospital for an examination between January 2010 and October 2020 and we analyzed sociodemographic, psychiatric and forensic characteristics of this population.

**Results:**

The number of people who have undergone a forensic psychiatric examination was 256. Three files were not usable due to lacking data. The offenders were men in 95.7% (242) of the cases.

Male inmates were found to be more likely to be re-incarcerated (p=0.029). The study showed that the level of education was an important factor in the recidivism of criminal acts (p=0.001) whilst no impact of marital status and employment were found (p=0.848; p=0.088).

Family history of psychiatric illness was significantly higher among recidivist (p=0.022).

Psychiatric factors associated with multiple convictions were the presence of a major psychiatric discord (p=0.0013), a personality disorder (p=0) or a substance use disorder (p=0.025).)

The reason of conviction was linked with criminal recidivism as violent offense were factors of incarceration (p=0) as well as the age at first conviction (p=0) and the number of anterior incarcerations (p=0).

**Conclusions:**

Our study underlines that predictive factors of criminality in general are also involved in recidivism among prisoners which is useful in establishing strategies of secondary but also primary prevention of violent crimes in particular.

**Disclosure of Interest:**

None Declared

